# Supplementary material for: Complexome profiling of the Chlamydomonas psb28 mutant reveals TEF5 as an early PSII assembly factor
Source: Plant Cell. 2025 Mar 17;37(6):koaf055. doi: 10.1093/plcell/koaf055 (PMC12164590; doi:10.1093/plcell/koaf055)
Supplement: koaf055_Supplementary_Data [file koaf055_supplementary_data.zip › psb28_interactive.zip!/plots/intensity/qualityPlots/peptides/_Cre06.g278179.t1.1.html]

### Cre06.g278179.t1.1

Trivial name:   
SeqCoverage [median]: 1.10  
Mass: 141.30 kDa  
  
 

| Sequence | MissedCleavage | Mass [Da] | wtIntensity | muIntensity | Score |
| --- | --- | --- | --- | --- | --- |
| AGITDAR | 0 | 702.37 | 0.00 | 8590.28 | 70.91 |
| SALADTPR | 0 | 829.43 | 113460.73 | 228913.01 | 111.01 |
|  |  |  | 113460.7 | 237503.3 |  |
